# The Influence of Tart Cherry (*Prunus cerasus, cv* Montmorency) Concentrate Supplementation for 3 Months on Cardiometabolic Risk Factors in Middle-Aged Adults: A Randomised, Placebo-Controlled Trial

**DOI:** 10.3390/nu13051417

**Published:** 2021-04-23

**Authors:** Rachel Kimble, Karen M. Keane, John K. Lodge, Glyn Howatson

**Affiliations:** 1 Faculty of Health and Life Sciences, Northumbria University, Newcastle-upon-Tyne NE1 8ST, UK; rachel.a.kimble@northumbria.ac.uk (R.K.); john.lodge@northumbria.ac.uk (J.K.L.); 2 Galway-Mayo Institute of Technology, School of Science and Computing, H91 T8NW Galway, Ireland; karen.keane@gmit.ie; 3 Water Research Group, School of Environmental Sciences and Development, Northwest University, Potchefstroom 2520, South Africa

**Keywords:** tart cherry, cardiovascular disease, vascular function, metabolic health

## Abstract

Background: Tart Montmorency cherries (MC) have been shown to be rich in anthocyanins and other phytochemicals known to have anti-inflammatory properties and influence pathways that might improve cardiometabolic health. However, there is limited evidence for the longer-term use of tart cherries on these indices. The aim of the current study was to investigate the influence of MC concentrate on cardiometabolic health indices following a 3-month supplementation period. Methods: Fifty middle-aged adults (34 males and 16 females; mean ± SD age: 48 ± 6 years and BMI: 27.6 ± 3.7 kg/m^2^) completed a randomised, placebo-controlled parallel study in which they either received MC or an isocaloric placebo. Participants drank 30 mL of their allocated treatment twice per day for 3 months. Vascular function (blood pressure [BP], heart rate [HR], pulse wave velocity and analysis [PWV/A], and flow mediated dilation [FMD]) as well as indices of metabolic health (insulin, glucose, lipid profiles, and high sensitivity C reactive protein) were measured following an overnight fast before and after the 3 months. Results: No effect of the intervention between the groups was observed for vascular function or metabolic health variables following the intervention (*p* > 0.05). However, MC concentrate was shown to be safe and well-tolerated and, importantly, did not have any deleterious effects on these outcomes. In conclusion, MC has no influence on cardiometabolic indices in middle-aged adults.

## 1. Introduction

Recent advances in technology and medicine have resulted in increased life expectancy and an aging population; it is now estimated that non-communicable diseases account for 71% of all deaths globally [1]. Cardiovascular disease (CVD) and type 2 diabetes combined are the primary cause of global mortality, increasing in prevalence with age [2]. Midlife risk factors have been proposed to underlie the development of these diseases, which evolve over years or decades before the emergence of clinical manifestations [3,4,5]. Thus, midlife has consistently been highlighted as a pivotal period for lifestyle interventions to improve health, reduce disease trajectory, and to promote healthy aging [6,7]. For example, middle-aged individuals who decrease their blood pressure (BP) to normal ranges (<120/<80 mmHg) have a significantly lower risk of CVD in their remaining lifetime compared with those with hypertension [8]. Furthermore, oxidative stress and inflammation are key mechanisms of endothelial dysfunction and arterial damage; thus, they are linked to type 2 diabetes [9] and CVD [4]. Consequently, there has been a research emphasis in natural dietary sources of antioxidants and foods with anti-inflammatory properties [10].

There is accumulating evidence that tart Montmorency cherries (MC) can positively impact cardiometabolic risk factors that include antioxidant [11], anti-inflammatory [12], and antihypertensive properties [13,14,15]. Tart MC are a relatively abundant source of anthocyanins [16]; the polyphenols responsible for the red-blue-purple pigmentation in fruit and vegetables. Previous work from our research group has shown that the higher intake of dietary anthocyanins is inversely associated with the risk of CVD mortality [17], and cherries have been reported to account for a considerable dietary intake of these compounds [18,19,20]. In vitro anthocyanin metabolites have been shown to interact with vascular smooth muscle cells [21] and upregulate endothelial nitric oxide synthase (eNOS) in endothelial cells [22]. More recently, the anthocyanin metabolites following blueberry intake were associated with improved endothelial function and nitric oxide (NO) bioavailability and were shown to influence genes involved in the regulation of cell adhesion, cell migration, inflammation, and cell differentiation processes, conferring the cardioprotective properties of these compounds [23].

Furthermore, MC are also rich in other phytochemicals (e.g., phenolic acids, flavonols, flavon-3-ols, melatonin, and carotenoids) that might have synergistic and additive effects on any bioactivities, including their antioxidant and anti-inflammatory actions [21,24]. Nonetheless, despite some promising epidemiological and in vitro studies suggesting a putative role for MC in cardiovascular and metabolic health, clinical trials have provided paradoxical and equivocal findings for any benefits associated with their intake. For example, relatively short-term (4–6 week) MC interventions failed to influence endothelial function, blood pressure, and cholesterol in middle-aged populations [11,12,25,26]. However, a longer-term (12 week) consumption of MC juice has been shown to reduce systolic BP and low-density lipoprotein (LDL) in older adults [14]. Moreover, Johnson and colleagues [27] demonstrated that 12, but not 6, weeks of MC reduce oxidised LDL in individuals with metabolic syndrome. To date, there has been no study investigating the longer-term influence of MC on cardiometabolic risk factors in a middle-aged population; however, based on recent findings, it was hypothesised that longer-term MC supplementation would improve vascular function and metabolic health parameters. Therefore, the aim of the current study was to investigate the influence of 3 months of MC concentrate supplementation on cardiometabolic health indices in middle-aged adults.

## 2. Materials and Methods

### 2.1. Participants

Non-smoking males and females between the ages of 40 to 60 years were recruited from Newcastle Upon Tyne and the local area of the city, using posters, email distributions, social media, and word of mouth. To be included in the study, participants must have reported to consume (on average) less than 5 servings of fruits and vegetables per day, did ≤4 h of moderate–vigorous physical activity per week, and had a ≥1 risk factor for type 2 diabetes. These risk factors included body mass index (BMI) >25 kg/m^2^; waist circumference >102 cm for males and >88 cm for females; family history of type 1 or type 2 diabetes; were a member of a type 2 diabetes high risk population (Aboriginal, Hispanic, Asian, South Asian, or African decent) or were hypertensive; >140/90 mmHG [28,29]. All participants were otherwise in apparent good health, as assessed by a health-screening questionnaire, not regularly taking medication (or stabilised ≥3 months, with no adverse symptoms) or antioxidant supplements and willing to report any changes in health status or medication during the study period.

Exclusion criteria were defined as a history of cardiometabolic disease, uncontrolled hypertension (SBP > 159 mmHg or DBP > 99 mmHg), gastrointestinal disease or malabsorption syndromes, reported changes in dietary or physical activity patterns within 3 months prior or intention to change during the study period, vegetarians, vegans or had known eating disorders, excessive alcohol intake, or a BMI ≥40 kg/m^2^. Additionally, participants who were pregnant or planning to become pregnant during the study, lactating, or initiating or changing a hormone replacement therapy regimen within 3 months of the start of the study were also excluded. This study was conducted in accordance with the Declaration of Helsinki and ratified by the University’s Research Ethics Committee prior to participants providing written, informed consent. This study was registered as a clinical trial with clinicaltrials.gov [NCT04021342].

### 2.2. Study Design

This study employed a randomised, double-blind, placebo-controlled, parallel design. After screening and recruitment, participants were familiarised with the testing equipment and procedures (visit 1). Following this, they were randomly assigned to receive either MC concentrate or an isocaloric placebo for 3 months using a computer-generated plan (https://www.randomization.com, accessed on 18 September 2018), stratified by sex. The study comprised of two experimental visits, following a minimum of a 7-day low anthocyanin run-in. Vascular function and metabolic health variables, as described below, were assessed at baseline (visit 2; pre-supplementation) and at 3 months (visit 3; post-supplementation). All experimental visits took place between 8:00 and 10:00 am and were preceded by an overnight fast (≥10 h). Participants were also asked to arrive hydrated and to avoid strenuous exercise, alcohol, and nutritional supplements 24 h and caffeine 12 h prior.

### 2.3. Dietary Intervention

A concentrated MC juice stored at 4 °C was used in this study. The concentrate was provided by Cherry Marketing Institute (USA). The participants were instructed to consume either 30 mL of MC concentrate diluted in 240 mL of water or the same volume of placebo twice daily; once in the morning and again in the evening. According to the manufacturers, this is the equivalent of ~180 cherries per day. Previous batch analysis by our laboratory, using the pH differential method and the modified Folin-Ciocalteu colorimetric method, showed that 60 mL of MC concentrate is the equivalent to 68–73.5 mg of anthocyanins and 160.8–178.8 mg of total phenolics, respectively [13,30]. The placebo was prepared by mixing unsweetened black cherry flavoured Kool-Aid (Kraft Foods, United States), dextrose (MyProtein Ltd., Northwich, UK), and fructose (Sports Supplements Ltd., Essex, UK) with bottled water to best match the calorie content of the MC concentrate (Energy = 102 kcal, volume = 30 mL, carbohydrates = 25.5 g, protein = 0 g and fat = 0 g). Additional lemon juice (which is not known to contain anthocyanins [31]), for tartness and preservation, and artificial food colouring were added so that the final product had the same visual properties [14,32]. The assigned treatment and a 30 mL measuring cup were supplied to the participants by a researcher (independent to the project) to ensure the study remained double-blinded. Compliance was measured by daily tick sheets and return of any unconsumed juice. To evaluate blinding efficacy, the participants were asked to guess which treatment they thought they had been taking, following the supplementation period.

Throughout the study, the participants were encouraged to maintain their habitual diet and exercise routines; however, they were asked to refrain from consuming cherries, cherry products, or any antioxidant supplements. Additionally, they were given verbal and written instructions to limit berry fruits, red grapes (including extracts and juices), and red wine (which are the highest contributors of dietary anthocyanins [18,19]) to ≤1 portion per day throughout the study period. The participants recorded their pre-evening meal before the first experimental visit and were asked to replicate this before the second experimental visit. To monitor dietary intake, the participants recorded a 3-day food and exercise diary (two consecutive weekdays and one weekend day), which was analysed retrospectively (Nutritics software, v5.09, Dublin, Ireland). They also completed the International Physical Activity Questionnaire (IPAQ) and a short form quality-of-life survey (SF-36 [33]) at the beginning and end of the study, to determine physical activity levels and tolerance to the intervention, respectively.

### 2.4. Anthropometry and Aerobic Capacity

Stature was measured to the nearest 0.1 cm, using a stadiometer, and body mass measured to the nearest 0.1 kg, using the same digital scale (Seca Scales 703, Seca Ltd. Birmingham, UK). Body composition (fat mass, fat percentage, and lean body mass) was measured by dual-energy X-ray absorptiometry (DXA; Hologic, Horizon, Manchester, UK). The scanner was calibrated before each assessment in accordance with the manufacturer’s guidelines, and participants were instructed to wear the same clothing for each visit. Adiposity distribution is also reported, where the android and gynoid regions of the trunk were defined by the Hologic APEX software (version 5.6) and the percentage fat of each region used to calculate the android/gynoid ratio. Exercise capacity (V̇O_2 max_) was also assessed before and after the intervention, using a sub-maximal cycle test; the Astrand-Rhyming single-stage, 6-min test [34,35]. All measurements were performed at baseline and on the return visit, 3 months later.

### 2.5. Vascular Function

Vascular function was assessed, as previously described, with participants in the supine position [36]. Briefly, BP and heart rate (HR) were measured using a non-invasive, automated vital signs monitor (Carescape V100; Dinamap), closely adhering to the guidelines specified by the European Society of Hypertension [37]. Peripheral BP measurements were taken in triplicate, each separated by 1 min [38], and the mean of the last 2 readings of systolic (SBP), diastolic (DBP), and HR were used for analysis (coefficient of variation; CV < 6%).

The pulse wave velocity and analysis (PWV/A) were determined through arterial tonometry, using the SphygmoCor CPV system (ScanMed Medical, Moreton in Marsh, UK). The PWV (in m/s) was determined between carotid and femoral sites with electrocardiogram gating. The PWA was recorded at the radial artery, and the corresponding augmentation index (AIx) was derived, using a generalised transfer function [39]. The AIx is influenced by heart rate [40]; therefore, the Aix, normalised for a standard heart rate of 75 bpm (AIx@75), was also measured. The SphygmoCor software (version 9.0, ScanMed Medical, Moreton in Marsh, UK) provides indices of quality control; if the measurement did not meet these control criteria, it was discarded and replaced by a new measurement. A minimum of two acceptable readings was obtained for both PWV and PWA and the average used for analysis.

Brachial artery flow mediated dilation (FMD) was acquired using ultrasonography (HDI-5000 SONO CT ultrasound machine; Philips Medical System). The resting baseline diameter was recorded for 1 min prior to occlusion of the artery, which was obtained by inflating a manual sphygmomanometer >50 mmHg above SBP. Occlusion of the artery was maintained for 5 min. The recording was resumed 1 min before deflating the cuff and 3 min thereafter. The average baseline resting diameter and peak diameter post-occlusion were determined, using semi-automated computer software (Brachial Analyzer; Medical Imaging Applications), which were used to calculate the percentage FMD.

### 2.6. Haematological Samples

Venous blood samples (~12 mL) were collected in lithium-heparin vacutainers (Becton, Dickinson and Company, Plymouth, UK). Due to sampling errors, samples were available for 40 participants (Cherry *n* = 19; Placebo *n* = 21). These were centrifuged at 3000× *g* (4 °C) for 10 min and the plasma aliquoted and stored at −80 °C to be analysed later. Plasma samples were analysed for cholesterol, high density lipoprotein (HDL) cholesterol, triglycerides, and glucose, using a colorimetric enzymatic method (CV < 3.7 %). Insulin was analysed using enzyme-linked immunosorbent assay (Mercodia, Sweden; CV = 12%). High sensitivity CRP (hs-CRP) was analysed using particle-enhanced immunoturbidimetric assay (CV = 7.8%). The non-HDL cholesterol was calculated as HDL subtracted from total cholesterol. The LDL cholesterol was calculated using the Friedewald equation [41]. The homeostatic model assessment of insulin resistance (HOMA-IR) was calculated, according to Matthews et al. [42], using the following formula:

HOMA-IR = (Fasting insulin × Fasting glucose)/405(1)

### 2.7. Power Calculation and Statistical Analysis

Power calculations were performed for the primary end point: change in SBP after 3 months of consumption. The power was based on the inter-individual variability for SBP measurement [36]; assuming an 80% power and a 0.05 significance level, the total number of subjects required to provide sufficient power to detect a 5 mmHg, a clinically meaningful amount [43] in a 2-arm, parallel study was estimated to be 50. A total of 60 participants were needed to allow for a 20% drop-out; however, only 56 were recruited before the study end.

All data were analysed using IBM SPSS statistics (v 26.0 for Windows; SPSS, Chicago, IL, USA), measures are reported as means ± standard deviation (SD) in tables and standard error (SE) in figures, unless otherwise stated. The normality of distribution for outcome measures was tested using the Shapiro Wilks test, and the assumptions were tested prior to analysis. Baseline characteristics were compared by Wilcoxon signed-rank test where data were continuous, and Chi-square test where data were categorical. Dietary, physical activity and SF-36 data were analysed using a two-way (treatment × time) analysis of variance (ANOVA). Treatment guess data were analysed by Chi-square test. The effect of the intervention on vascular function and metabolic health variables was evaluated using a one-way analysis of covariance (ANCOVA), adjusted for baseline [44]. Additional covariates of sex and use of medication were added into the analysis for the primary outcome (vascular function). For blood samples, where values fell below the limits of detection, the sensitivity threshold of the assay was used to maintain participant numbers. The hs-CRP, HOMA-IR, and insulin values were non-normally distributed; thus, they were log transformed before analysis. Sidak adjusted post hoc comparisons were then carried out between cherry juice and placebo, as appropriate.

## 3. Results

**Figure 1:**
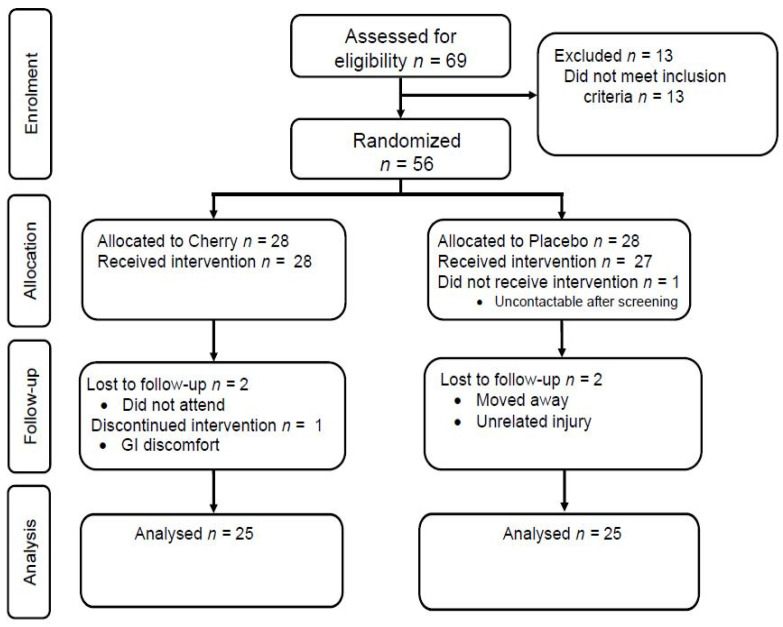
Consort flow diagram of participant enrolment and analysis in the study.

A total of 56 individuals were enrolled in the study and randomised to the intervention (Figure 1). There was no difference between the group characteristics at baseline (Table 1). Three participants from each group did not complete the study, as shown in Figure 1. One participant in the cherry group discontinued the juice and withdrew from the study due to gastrointestinal discomfort and bloating. The treatments were otherwise well-tolerated, as suggested by the SF-36, which showed no treatment, time, or interaction differences between the groups (data not shown). The mean (± SD) self-reported compliance (as assessed by tick sheets) was 94 ± 9% and 98 ± 4% in the cherry and placebo group, respectively. Five participants (20%) correctly guessed that they were in the placebo group; however, the Chi-square test suggested successful blinding (*p* = 0.386).

### 3.1. Physical Activity, Diet, and Body Composition

There was no treatment, time, or treatment × time interaction effects observed for physical activity, sitting time, or exercise capacity (Table 2). The analysis of 3-day diet records showed that there were no differences between the two groups for the mean intake of total energy, fat, or saturated fat intake. Protein intake showed a treatment × time interaction (F = 7.8, *p* = 0.011). Planned post hoc tests revealed that protein intake at baseline was, on average, 12 g higher in the placebo group compared with the cherry group (F = 6.7, *p* = 0.016). There was also an increase in carbohydrate (36 g) intake at 3 months in both groups, with a main effect of time (F = 17.3, *p* < 0.001). Body mass (F = 8.5, *p* = 0.007), BMI (F = 12.6, *p* = 0.002), and fat mass (F = 4.8, *p* = 0.040) increased relative to baseline in the placebo group, main effect of time. Fat mass (F = 7.7, *p* = 0.011) and fat percentage (F = 4.38, *p* = 0.047) also increased after 3 months in the cherry group. There was no treatment effect observed at baseline or after 3 months for body mass. There was no treatment, time, or treatment × time interaction effects between lean mass or android/gynoid ratio (Table 2).

### 3.2. Influence of MC on Vascular Function

**Figure 2:**
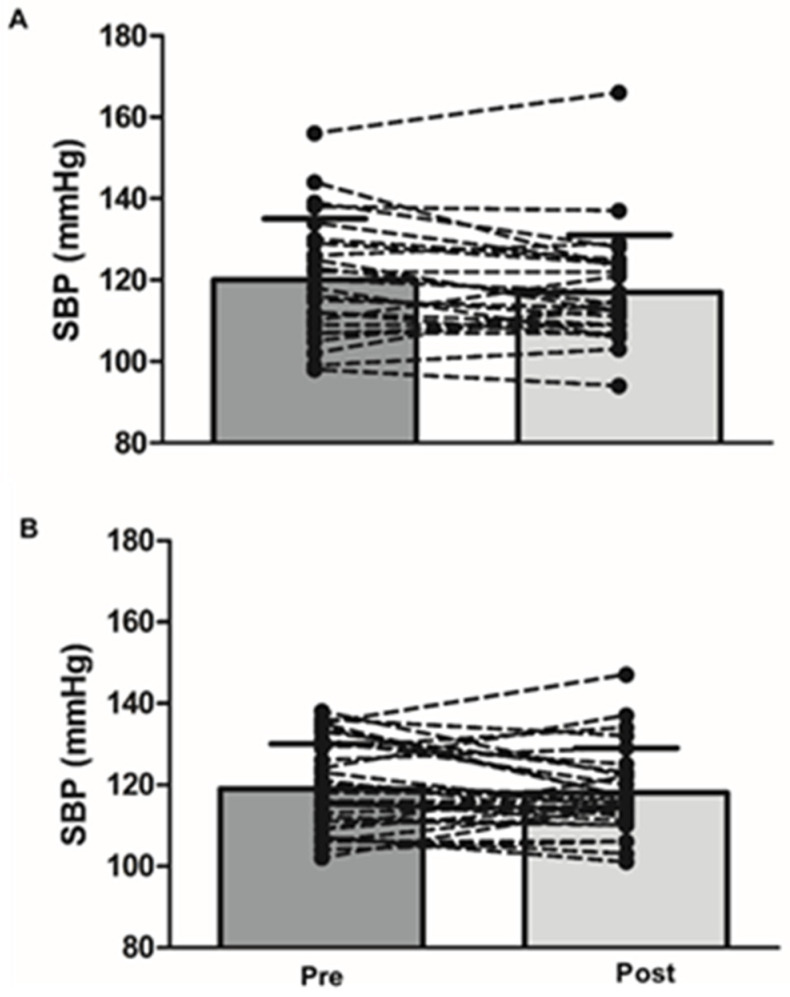
Systolic blood pressure before (left) and after 3-month supplementation (right) with cherry juice (**A**) and placebo (**B**).

After adjusting the baseline (pre-treatment) values, sex, and medication, there were no group differences between MC and placebo at 3 months for SBP (117 ± 14 vs. 118 ± 11 mmHg; *p* = 0.8; Figure 2). After 3 months, there was also no difference for DBP, HR, arterial stiffness (PWV, AIx and AIx@75), or endothelial function (FMD), compared to the placebo (Table 3).

### 3.3. Influence of MC on Metabolic Health Indices

After adjusting the baseline (pre-treatment) values, there were no group differences between cherry juice and placebo for lipid profiles, insulin, glucose, HOMA-IR, or hs-CRP (Table 4).

## 4. Discussion

To date, this is the largest and longest duration study to determine the influence of MC on vascular function and metabolic health in free-living, middle-aged adults. The primary aim was to assess any change in vascular function, specifically SBP, which was shown to be modulated in previous work [14,30]. However, based on the vascular function variables measured in the current study, and, contrary to the hypothesis, there was no effect on BP, endothelial function, or arterial stiffness (Table 3). While the reasons for this could be manifold, it might be explained by the kinetics of tart cherry phenolics. We and others have previously shown that tart cherry anthocyanin metabolites peak in the plasma within 1–2 h, a time course which coincides with the greatest reductions in postprandial SBP [13,15,45]. Moreover, there is a rapid clearance in these metabolites, with SBP returning to basal within 3–4 h [13,15]. In the current study, vascular function was measured after an overnight fast; therefore, the peak vasomodulatory properties of the MC might have been missed. This represents a limitation of the current study, as acute responses were not investigated; however, the study was ultimately interested in the cumulative influence of MC consumption. In a recent addition to the literature, Desai and colleagues [46] demonstrated that a 6-day supplementation with 30 mL MC had no influence on fasted or post-bolus laboratory SBP; however, it did influence a 24 h ambulatory SBP after the 7th day. These data could suggest a first dose phenomenon similar to that of antihypertensive medications, in that the large fall in SBP is as an initial response to the peak tart cherry phenolics but the magnitude of which is less pronounced over time [47].

In contrast to the current study, two randomised controlled studies have shown that tart [14] and sweet cherry juice [48] reduce SBP in older adults, following a 12-week supplementation. The reason for sustained changes in vascular function, following chronic anthocyanin and other polyphenol supplementation, are currently unknown; however, they are possibly mediated via the modulation of the microbiome and complex gene expression alterations [23,49,50]. Therefore, the genetic changes and reductions in microbial diversity that occur in older adults [51,52] might explain why the aforementioned studies [14,48] found a beneficial effect of MC. However, in the current study, middle-aged adults likely have higher numbers and diversity of protective microbial species [53,54]; hence, they are less amenable to these changes [25]. Other potential reasons for these discrepancies could be due to inter-individual differences in the response or initial vascular function of the cohort. A recent review of the factors that influence the efficacy of anthocyanins on BP regulation highlighted that baseline BP was an important factor, with changes only evident in those with an elevated initial BP [55]. Despite recruiting middle-aged individuals with additional risk-factors for CVD, the participants in the current study were either pre-symptomatic or had controlled hypertension, thus had BP readings within the normal range, whereas the older adults had higher BP at baseline. Moreover, the data in the present study are in line with those of the others that longer tart cherry supplementation does not influence resting SBP in normotensive individuals [11,56], even those with increased CVD risk [12,27]; this suggests that the latter reason is the most probable one for the disparity between the studies.

Endothelial dysfunction and arterial stiffness are indicators of subclinical atherosclerosis and precede hypertension; thus, they are major risk factors for CVD [57], though are also modifiable by diet [3]. Here, we found no noteworthy changes in FMD, PWV, or PWA. However, it should be acknowledged that the participants in the current study were on medications, and these could have had an influence on these variables; although they were included in the statistical analysis, they could have confounded the results. Nonetheless, according to the HSE [58], nearly half (48%) of the adults in England are regularly taking at least one prescription drug; therefore, the cohort in this study has general applicability to the wider population. Importantly, our findings are consistent with the literature. For example, Lynn and colleagues [11] reported no effect on brachial-knee PWV in healthy middle-aged adults, following a 6-week supplementation with 30 mL MC concentrate, in an open-labelled randomised controlled trial. Similarly, Johnson et al. [27] reported no effect of a 12-week bi-daily MC consumption on Aix, when corrected for a HR of 75 beats/min or PWV, in individuals with metabolic syndrome. Moreover, Aboo-Bakker et al. [26] demonstrated that a 4-week supplementation of MC powder (256 mg/day anthocyanins split into two doses, morning and evening) did not improve resting FMD; however, it did restore FMD and enhance the recovery of plasma nitrite following occlusion-induced ischemia reperfusion. Likewise, in clinical populations with greater impairment to vascular function, anthocyanin-rich foods have been shown to improve these parameters [59,60,61]. Therefore, while early intervention in at-risk individuals (such as those in the current study) remains a research priority in reversing or reducing the disease risk trajectory preventing CVD, it is plausible that improvements following MC intake in these individuals might require more longitudinal observations. Those with pathological conditions might benefit more from MC supplementation due to the systemic pro-inflammatory and pro-oxidative state associated with these [62]. Likewise, those with hypertension or other cardiovascular dysfunctions might benefit more. Therefore, future work could focus on these sorts of pathology to examine this idea.

The current study also identified no changes in the markers of metabolic health or inflammation following the intervention (Table 4). These findings are not alone, given that tart cherries have failed to influence cholesterol [11], insulin concentrations/resistance [63], and markers of systemic inflammation [25] in similar populations. In the current study, the levels of these markers before the intervention were all within normal ranges and thus, similar to BP, less likely to benefit from an intervention, relative to those with elevated baseline values, i.e., older individuals and those with metabolic or inflammatory conditions [12,14,15,64]. Notably, despite the relatively high sugar content of the concentrate and subsequent increase in carbohydrate intake over the intervention, there were no deleterious influences on these outcomes in response to the additional glycaemic stress [65]. Moreover, there is epidemiological evidence that suggests higher intake of dietary anthocyanins is associated with reduced inflammation, insulin resistance, risk of type 2 diabetes, and CVD [17,66,67]; therefore, again, we cannot rule out the influence of longer study durations.

It should be acknowledged that there was a marked effect of the intervention on body composition in both groups. After 3 months, both body mass and BMI were higher in the placebo group, whereas fat percentage was higher in the cherry group and fat mass had increased in both groups (Table 2). In the current study, the MC concentrate was given as an adjunct to the diet, as previous studies had suggested little influence on body composition [12,27]. A limitation of the present study is that dietary records and IPAQ were only collected at the beginning and end of the study, as opposed to throughout; although total energy intake, physical activity, or exercise capacity did not appear to change, we cannot rule out that changes may be seasonal and un-related to the intervention [68]. However, future studies should adjust diet to accommodate calories from juice, particularly since interventions demonstrate that energy from beverages leads to little dietary compensation and results in weight gain because beverages possess poor satiating properties compared to their solid equivalents [69]. Moreover, the changes in body composition following MC intake are not in isolation. For example, Chai and colleagues [14] reported a higher BMI (1.06 kg/m^2^) in those consuming MC concentrate for 12 weeks. More recently, Dodier et al. [70] reported an increase in body mass and BMI, following the intake of 240 mL/day of MC for 90 days, warranting careful consideration in future research designs.

Due to the low number of adverse events, good compliance levels reported, and no effect on quality-of-life indices, it is reasonable to suggest that cherry juice is a safe and tolerable intervention, although future studies should consider dietary modification to accommodate for the increase in Kcal from the juice. This study has several strengths such that it was successfully blinded, sufficiently powered, of a longer-term duration, and well-controlled, compared to other studies of a similar nature [11,14,15,62,71]. However, there are other limitations that warrant discussion. Firstly, compliance to the intervention was self-reported; as these were free-living adults, there was no control over whether they adhered to the intervention, how they stored the concentrate, or when they consumed the MC. Secondly, anthocyanin content is reported based on previous analysis, which was conducted in our laboratory; however, batch-to-batch variation can naturally occur due to growing conditions (e.g., soil, use of fertiliser, time of year, weather), storage, and processing. Additionally, the lack of specific analytics [72] in the current study needs to be considered while interpreting the findings. Lastly, BP was measured in the laboratory, which was shown to be reliable; however, a 24 h ambulatory BP could be more advantageous in establishing small changes in BP following MC supplementation [46], due to the large number of readings and avoidance of ‘white-coat hypertension’.

## 5. Conclusions

In conclusion the current study found no effect of a 3-month MC concentrate on vascular function and metabolic health indices. Given the current interest in the facilitation of healthy aging, investigation into the intake of anthocyanin-rich foods such as MC, in pre-symptomatic and at-risk individuals, are important as a means to preserve cardiometabolic health, although more longitudinal observations might be required. Future studies should consider utilising populations with similar risk factors (e.g., hypertension) and impairments in vascular and metabolic function at baseline, which could provide a suitable population to examine the potential efficacy of foods rich in these compounds. 

## Figures and Tables

**Table 1:** Baseline characteristics (Mean ± SD).

Characteristic	All (*n* = 56)	Cherry (*n* = 28)	Placebo (*n* = 28)	*p*-Value
Age (y)	48 ± 6	49 ± 6	47 ± 6	0.160
Sex (m/f)	37/19	19/9	18/10	0.778
Stature (cm)	173.1 ± 8.8	173.7 ± 8.9	172.4 ± 9.0	0.494
Body Mass (kg)	81.8 ± 12.9	81.7 ± 14.0	82.0 ± 11.9	0.793
BMI (kg/m^2^)	27.3 ± 3.7	27.0 ± 3.8	27.4 ± 3.7	0.569
*Ethnicity* (*n*; %)				0.368
White	54 (96.4)	27 (96.4)	27 (96.4)	
*Education* (*n*; %)				0.798
Less Than High School	−	−	−	
High School or Equivalent	24 (43)	11 (39)	13 (46)	
Bachelor’s Degree	19 (34)	9 (32)	10 (36)	
Postgraduate Degree	13 (23)	8 (29)	5 (18)	
Medication (*n*; %)	17 (30)	10 (36)	7 (25)	0.771

**Table 2:** Physical activity, diet, and body composition during the study.

	Cherry	Placebo	ANOVA		
			Treatment	Time	Interaction
METs (min/week)					
Baseline	2462 ± 2038	1953 ± 1527	0.267	0.959	0.961
3 months	2464 ± 2336	1978 ± 1217			
Sitting time (h)					
Baseline	6.6 ± 3.1	6.7 ± 3.0	0.856	0.852	0.486
3 months	6.7 ± 2.8	6.3 ± 3.0			
V̇O_2 max_ (mL·kg^−1^·min^−1^)					
Baseline	35.9 ± 11.1	37.3 ± 7.5	0.319	0.187	0.386
3 months	33.7 ± 9.6	37.1 ± 6.2			
Energy (Kcal)					
Baseline	1921 ± 340	1896 ± 439	0.997	0.212	0.673
3 months	1977 ± 439	2001 ± 423			
Carbohydrates (g)					
Baseline	208.8 ± 38.6	196.1 ± 47.3	0.671	<0.001	0.350
3 months	237.3 ± 70.0 ^#^	239.6 ± 66.7 ^#^			
Fat (g)					
Baseline	76.5 ± 18.5	78.4 ± 24.2	0.681	0.231	0.999
3 months	72.1 ± 18.1	74.1 ± 21.2			
Saturated fat (g)					
Baseline	28.2 ± 8.6	27.5 ± 8.0	0.849	0.224	0.836
3 months	25.8 ± 8.3	25.7 ± 10.6			
Protein (g)					
Baseline	80.2 ± 21.9	92.3 ± 16.9 *	0.193	0.051	0.006
3 months	80.3 ± 15.7	78.5 ± 19.3 ^#^			
Body mass (kg)					
Baseline	82.8 ± 13.9	82.4 ± 12.3	0.968	0.007	0.048
3 months	83.4 ± 14.2	84.1 ± 13.2 ^#^			
BMI (kg/m^2^)					
Baseline	27.3 ± 3.8	27.5 ± 3.8	0.757	0.001	0.057
3 months	27.5 ± 3.8	28.1 ± 4.0 ^#^			
Body fat (%)					
Baseline	37.1 ± 7.9	36.1 ± 6.8	0.642	0.031	0.862
3 months	37.7 ± 6.8 ^#^	36.7 ± 6.8			
Fat mass (kg)					
Baseline	30.2 ± 9.1	29.6 ± 7.1	0.856	0.007	0.691
3 months	31.1 ± 9.5 ^#^	30.7 ± 7.5 ^#^			
Lean mass (kg)					
Baseline	48.1 ± 9.4	49.8 ± 8.5	0.360	0.248	0.199
3 months	48.1 ± 8.5	50.4 ± 9.2			
Android/gynoid ratio					
Baseline	1.09 ± 0.17	1.13 ± 0.18	0.394	0.403	0.529
3 months	1.08 ± 0.17	1.13 ± 0.18			

Mean ± SD; body mass index (BMI); ^#^ significantly different from baseline; * significantly different between groups (*p* < 0.05).

**Table 3:** Influence of tart Montmorency cherries on vascular function compared to a placebo.

	Cherry Juice	Placebo	ANCOVA Adjusted for Baseline			Adjusted for Baseline, Sex and Medication		
			Difference (95% CI)	F	*p*-Value	Difference (95% CI)	F	*p*-Value
SBP (mmHg)								
Baseline	120 ± 15	119 ± 11	−0.5 (−4.9, 3.8)	0.056	0.814	−0.6 (−5.1, 3.9)	0.079	0.780
3 months	117 ± 14	118 ± 11						
DBP (mmHg)								
Baseline	73 ± 10	73 ± 8	−0.4 (−3.0, 2.3)	0.086	0.770	−0.5 (−3.1, 2.2)	0.127	0.723
3 months	73 ± 9	73 ± 8						
HR (BPM)								
Baseline	59 ± 11	59 ± 10	−0.3 (−3.7, 3.1)	0.033	0.858	−0.2 (−3.6, 3.2)	0.014	0.908
3 months	59 ± 12	59 ± 10						
PWV (m/s)								
Baseline	6.7 ± 1.0	6.4 ± 0.8	0.3 (−0.3, 0.8)	1.051	0.312	0.2 (−0.2, 0.7)	0.967	0.332
3 months	6.8 ± 1.3	6.2 ± 0.8						
AIx (%)								
Baseline	22.1 ± 8.9	17.8 ± 11.4	0.3 (−3.6, 4.2)	0.021	0.886	0.3 (−3.6, 4.2)	0.022	0.884
3 months	20.4 ± 9.6	17.0 ± 10.0						
AIx@75 (%)								
Baseline	13.4 ± 8.1	9.6 ± 12.9	0.02 (−3.6, 3.6)	<0.001	0.991	−0.06 (−3.6, 3.5)	0.001	0.937
3 months	12.3 ± 9.2	9.3 ± 11.0						
FMD (%)								
Baseline	8.3 ± 3.5	9.3 ± 3.5	1.1 (−1.1, 3.3)	0.972	0.330	1.2 (−1.0, 3.3)	1.256	0.269
3 months	9.7 ± 3.5	9.0 ± 4.0						

Mean ± SD; Abbreviations; augmentation index (AIx); AIx normalised for a heart rate of 75 bpm (AIx@75); diastolic blood pressure (DBP); flow-mediated dilation (FMD); heart rate (HR); pulse wave velocity (PWV).

**Table 4:** Influence of tart Montmorency cherries on metabolic health indices.

	Cherry (*n* = 19)	Placebo (*n* = 21)	ANCOVA Adjusted for Baseline		
			Difference (95% CI)	F	*p*-Value
Insulin (pmol/L)					
Baseline	20.3 ± 15.4	19.6 ± 10.2	−0.01 (−0.18, 0.15)	0.020	0.888
3 months	17.8 ± 10.0	19.0 ± 13.0			
Glucose (mmol/L)					
Baseline	5.4 ± 0.5	5.5 ± 0.5	−0.01 (−0.21, 0.18)	0.016	0.899
3 months	5.4 ± 0.5	5.4 ± 0.3			
HOMA-IR					
Baseline	0.7 ± 0.6	0.7 ± 0.4	0.04 (−0.13, 0.21)	0.239	0.629
3 months	0.7 ± 0.4	0.7 ± 0.5			
hs-CRP (mg/L)					
Baseline	1.6 ± 2.3	1.2 ± 1.2	−0.003 (−0.14, 0.14)	0.111	0.741
3 months	1.4 ± 1.7	1.2 ± 1.0			
Triglycerides (mmol/L)					
Baseline	1.2 ± 0.6	1.2 ± 0.7	−0.06 (−0.29, 0.18)	0.241	0.627
3 months	1.2 ± 0.7	1.3 ± 0.7			
Cholesterol (mmol/L)					
Baseline	5.3 ± 1.2	5.0 ± 1.0	−0.15 (−0.51, 0.21)	0.728	0.399
3 months	5.2 ± 1.1	5.1 ± 0.9			
LDL Cholesterol (mmol/L)					
Baseline	3.2 ± 1.0	3.0 ± 1.0	−0.09 (−0.38, 0.20)	0.411	0.525
3 months	3.1 ± 1.0	3.1 ± 1.0			
HDL Cholesterol (mmol/L)					
Baseline	1.6 ± 0.3	1.5 ± 0.4	−0.02 (−0.15, 0.12)	0.061	0.806
3 months	1.5 ± 0.4	1.5 ± 0.4			
Non-HDL Cholesterol (mmol/L)					
Baseline	3.7 ± 1.2	3.5 ± 1.2	−0.17 (−0.49, 0.16)	1.08	0.305
3 months	3.7 ± 1.1	3.6 ± 1.1			
Total/HDL Cholesterol Ratio					
Baseline	3.5 ± 1.0	3.7 ± 1.3	−0.076, −0.41, 0.26)	0.209	0.650
3 months	3.6 ± 1.2	3.8 ± 1.4			

Mean ± SD. Abbreviations: high-density lipoproteins (HDL); homeostatic model assessment of insulin resistance (HOMA-IR); high-sensitivity C-reactive protein (hs-CRP) log transformed before analysis; raw values are presented.

## Data Availability

Anonymised data described in the manuscript, code book, and analytic code will be made available upon reasonable request to the Principal Investigator.
